# 4,4,5,5-Tetra­methyl-2-[4-(2-pyrid­yl)phen­yl]-3,4-dihydro­imidazole-1-oxyl-3-oxide

**DOI:** 10.1107/S1600536809013221

**Published:** 2009-04-10

**Authors:** Xiang-Yang Qin, Ping-An Wang, Xiao-Li Sun

**Affiliations:** a Department of Chemistry, School of Pharmacy, Fourth Military Medical University, Changle West Road 17, 710032 Xi-An, People’s Republic of China

## Abstract

In the title compound, C_18_H_20_N_3_O_2_, the pyridine and phenyl rings are coplanar [dihedral angle = 3.5 (3)°]. The phenyl ring makes a dihedral angle of 29.6 (1)° with the imidazole ring. The crystal structure is stabilized by inter­molecular C—H⋯O hydrogen bonds.

## Related literature

For the preparation of the title compound see: Ullman *et al.* (1974 ▶). For recent synthetic use of the title compound and its derivatives, see: Li *et al.* (2009 ▶); Xu *et al.* (2008 ▶); Masuda *et al.* (2009 ▶); Train *et al.* (2009 ▶).
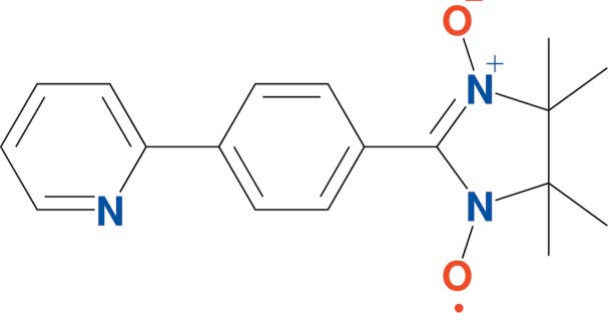


## Experimental

### 

#### Crystal data

C_18_H_20_N_3_O_2_
*M* *_r_* = 310.37
Monoclinic, 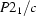

*a* = 8.5150 (17) Å
*b* = 22.286 (5) Å
*c* = 9.1360 (18) Å
β = 109.45 (3)°
*V* = 1634.8 (6) Å^3^
*Z* = 4
Mo *K*α radiation
μ = 0.08 mm^−1^
*T* = 293 K
0.20 × 0.20 × 0.20 mm

#### Data collection

Bruker SMART APEX CCD diffractometer
Absorption correction: multi-scan (**SADABS**; Bruker, 2005 ▶) *T*_min_ = 0.983, *T*_max_ = 0.983
12953 measured reflections
2819 independent reflections
1896 reflections with *I* > 2σ(*I*)
*R*_int_ = 0.063

#### Refinement

*R*[*F* ^2^ > 2σ(*F* ^2^)] = 0.070
*wR*(*F* ^2^) = 0.146
*S* = 1.09
2819 reflections
212 parameters
H-atom parameters constrained
Δρ_max_ = 0.21 e Å^−3^
Δρ_min_ = −0.20 e Å^−3^

### 

Data collection: *APEX2* (Bruker, 2007 ▶); cell refinement: *SAINT* (Bruker, 2007 ▶); data reduction: *SAINT*; program(s) used to solve structure: *SHELXS97* (Sheldrick, 2008 ▶); program(s) used to refine structure: *SHELXL97* (Sheldrick, 2008 ▶); molecular graphics: *SHELXTL* (Sheldrick, 2008 ▶); software used to prepare material for publication: *Mercury* (Macrae *et al.*, 2006 ▶) and *CAMERON* (Watkin *et al.*, 1996 ▶).

## Supplementary Material

Crystal structure: contains datablocks I, global. DOI: 10.1107/S1600536809013221/bt2927sup1.cif

Structure factors: contains datablocks I. DOI: 10.1107/S1600536809013221/bt2927Isup2.hkl

Additional supplementary materials:  crystallographic information; 3D view; checkCIF report

## Figures and Tables

**Table 1:** Hydrogen-bond geometry (Å, °)

*D*—H⋯*A*	*D*—H	H⋯*A*	*D*⋯*A*	*D*—H⋯*A*
C12—H12⋯O2^i^	0.93	2.43	3.322 (4)	161

Symmetry code: (i) 
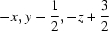
.

**Fig. 1.**
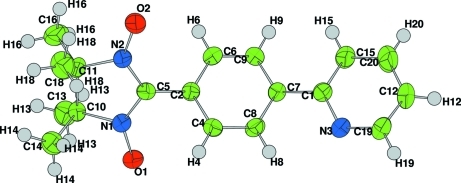
The molecular structure of the title compound, showing the atomic labelling scheme. The displacement ellipsoids are drawn at the 50% probability level. The hydrogen atoms are drawn as spheres of arbitrary radius.

**Fig. 2.**
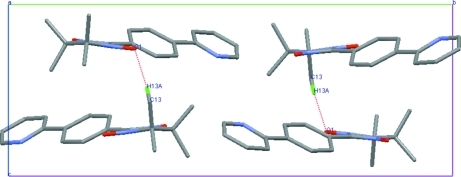
The packing of the title molecules, viewed down the a axis. Dotted lines indicate hydrogen bonds.

## supplementary crystallographic information

### Comment

The title radical compound was obtained the oxidation of 4,4,5,5- tetramethyl-2-(4-(pyridin-2-yl)phenyl)imidazolidine-1,3-diol, which was prepared by the condensation of 4-(pyridin-2-yl)benzaldehyde with 2,3-Dimethyl-2,3-bis(hydroxyl-amino)butane. The title compound was used for coordination with many metal cations, such as Mn^2+^, Cu^2+^, Ni^2+^ and Zn^2+^, in order to form some molecule-based magnetic materials (Train *et al.*, 2009; Masuda *et al.*, 2009).

In the crystal structure of the title compound, the pyridine ring and the phenyl ring are in one same plane, and this aromatic ring system is twisted with respect to the imidazole ring with a dihedral angle of 29.6 (1)°, and the packing of molecules in the crystal structure is stabilized by intermolecular C—H···O hydrogen bonds. In the imidazole ring, the length of N1—O1 is 1.284 (3) Å, while the length of N2—O2 is 1.274 (3) Å.

### Experimental

The title compound (I) was prepared according to the method reported by Ullman *et al.* (1974). 2,3-Dimethyl-2,3-bis(hydroxylamino)butane (1.48 g, 10.0 mmol) and 4-(pyridin-2-yl)benzaldehyde (1.83 g, 10.0 mmol) were dissolved in a methanol solution (20.0 ml), which was stirred for 3 h at room temperature, and then filtered, the cake was washed by methanol (5.0 ml) for twice. This product was dried under vaccum, then, it was suspended in dichloromethane (100.0 ml) and this reaction mixture was cooled at ice bath for 10 min, the water solution (30.0 ml) of NaIO_4_ (1.7 g,) was added dropwise to the above suspension and stirred for 20 min at this temperature, the organic layer was seperated and the aqueous phase was extracted by dichloromethane (30.0 ml) for twice. The combined organic layer was dried over Na_2_SO_4_ and the solvent was removed to give a dark blue residue which was purified by a flash column chromatography (eluent, ether and petroleum ether, the ratio of volume is 4 to 1) to yield the title compound (I) as a dark blue powder. Single crystals of (I) were obtained from the mixed solution of *n*-heptane and dichloromethane (the ratio of volume is 4 to 1).

### Refinement

In both structures all the H atoms were discernible in the difference Fourier maps. However, they were constrained by riding model approximation. *C*—H_methyl_=0.96 Å; *C*—H_aryl_=0.93 Å; *U*_iso_H_methyl_ and *U*_iso_H_aryl_ are 1.5 U _eq_(C) and 1.2 U _eq_(C), respectively.

### Crystal data

**Table:**

C_18_H_20_N_3_O_2_	*F*(000) = 660
*M**_r_* = 310.37	*D*_x_ = 1.261 Mg m^−^^3^
Monoclinic, *P*2_1_/*c*	Mo *K*α radiation, λ = 0.71073 Å
*a* = 8.5150 (17) Å	Cell parameters from 2819 reflections
*b* = 22.286 (5) Å	θ = 3.0–25.0°
*c* = 9.1360 (18) Å	µ = 0.08 mm^−^^1^
β = 109.45 (3)°	*T* = 293 K
*V* = 1634.8 (6) Å^3^	Block, blue
*Z* = 4	0.20 × 0.20 × 0.20 mm

### Data collection

**Table:**

Bruker SMART APEX CCD diffractometer	2819 independent reflections
Radiation source: fine-focus sealed tube	1896 reflections with *I* > 2σ(*I*)
graphite	*R*_int_ = 0.063
Detector resolution: 0 pixels mm^-1^	θ_max_ = 25.0°, θ_min_ = 3.0°
ω scans	*h* = −10→10
Absorption correction: multi-scan (*SADABS*; Bruker, 2005)	*k* = −26→26
*T*_min_ = 0.983, *T*_max_ = 0.983	*l* = −10→10
12953 measured reflections	

### Refinement

**Table:**

Refinement on *F*^2^	Primary atom site location: structure-invariant direct methods
Least-squares matrix: full	Secondary atom site location: difference Fourier map
*R*[*F*^2^ > 2σ(*F*^2^)] = 0.070	Hydrogen site location: inferred from neighbouring sites
*wR*(*F*^2^) = 0.146	H-atom parameters constrained
*S* = 1.09	*w* = 1/[σ^2^(*F*_o_^2^) + (0.0512*P*)^2^ + 0.5834*P*] where *P* = (*F*_o_^2^ + 2*F*_c_^2^)/3
2819 reflections	(Δ/σ)_max_ < 0.001
212 parameters	Δρ_max_ = 0.21 e Å^−^^3^
0 restraints	Δρ_min_ = −0.20 e Å^−^^3^

### Special details

**Table:**

Geometry. All e.s.d.'s (except the e.s.d. in the dihedral angle between two l.s. planes) are estimated using the full covariance matrix. The cell e.s.d.'s are taken into account individually in the estimation of e.s.d.'s in distances, angles and torsion angles; correlations between e.s.d.'s in cell parameters are only used when they are defined by crystal symmetry. An approximate (isotropic) treatment of cell e.s.d.'s is used for estimating e.s.d.'s involving l.s. planes.
Refinement. Refinement of *F*^2^ against ALL reflections. The weighted *R*-factor *wR* and goodness of fit *S* are based on *F*^2^, conventional *R*-factors *R* are based on *F*, with *F* set to zero for negative *F*^2^. The threshold expression of *F*^2^ > σ(*F*^2^) is used only for calculating *R*-factors(gt) *etc*. and is not relevant to the choice of reflections for refinement. *R*-factors based on *F*^2^ are statistically about twice as large as those based on *F*, and *R*- factors based on ALL data will be even larger.

### Fractional atomic coordinates and isotropic or equivalent isotropic displacement parameters (Å^2^)

**Table:**

	*x*	*y*	*z*	*U*_iso_*/*U*_eq_	
C1	0.0559 (3)	0.57868 (12)	0.7773 (3)	0.0406 (7)	
C2	0.3900 (3)	0.72221 (12)	0.7712 (3)	0.0370 (6)	
C4	0.3958 (3)	0.66754 (12)	0.6990 (3)	0.0432 (7)	
H4	0.4731	0.6620	0.6483	0.052*	
C5	0.5033 (3)	0.77122 (11)	0.7688 (3)	0.0377 (6)	
C6	0.2755 (3)	0.72855 (12)	0.8480 (3)	0.0469 (7)	
H6	0.2695	0.7646	0.8972	0.056*	
C7	0.1736 (3)	0.62798 (11)	0.7782 (3)	0.0365 (6)	
C8	0.2889 (3)	0.62182 (12)	0.7019 (3)	0.0440 (7)	
H8	0.2939	0.5859	0.6516	0.053*	
C9	0.1706 (3)	0.68223 (12)	0.8522 (3)	0.0482 (8)	
H9	0.0959	0.6873	0.9058	0.058*	
C10	0.7488 (3)	0.82333 (12)	0.7781 (3)	0.0422 (7)	
C11	0.6108 (3)	0.86972 (12)	0.7722 (3)	0.0412 (7)	
C12	−0.1592 (4)	0.48645 (15)	0.7597 (4)	0.0699 (10)	
H12	−0.2267	0.4533	0.7561	0.084*	
C13	0.8852 (4)	0.81846 (14)	0.9347 (4)	0.0612 (9)	
H13A	0.8362	0.8120	1.0140	0.092*	
H13B	0.9490	0.8549	0.9558	0.092*	
H13C	0.9569	0.7854	0.9333	0.092*	
C14	0.8263 (4)	0.82919 (15)	0.6510 (4)	0.0671 (10)	
H14A	0.9077	0.7981	0.6628	0.101*	
H14B	0.8789	0.8677	0.6585	0.101*	
H14C	0.7411	0.8254	0.5514	0.101*	
C15	−0.0669 (5)	0.58456 (16)	0.8416 (5)	0.0849 (13)	
H15	−0.0773	0.6201	0.8908	0.102*	
C16	0.6523 (4)	0.91381 (15)	0.9054 (4)	0.0726 (11)	
H16A	0.5576	0.9390	0.8945	0.109*	
H16B	0.7450	0.9382	0.9045	0.109*	
H16C	0.6808	0.8923	1.0017	0.109*	
C18	0.5432 (4)	0.90355 (16)	0.6188 (4)	0.0720 (10)	
H18A	0.5157	0.8755	0.5343	0.108*	
H18B	0.6262	0.9310	0.6094	0.108*	
H18C	0.4452	0.9254	0.6161	0.108*	
C19	−0.0414 (4)	0.48541 (14)	0.6919 (4)	0.0678 (10)	
H19	−0.0351	0.4514	0.6352	0.081*	
C20	−0.1762 (6)	0.53756 (18)	0.8335 (6)	0.1057 (16)	
H20	−0.2595	0.5410	0.8779	0.127*	
N1	0.6539 (3)	0.76539 (10)	0.7555 (3)	0.0437 (6)	
N2	0.4734 (3)	0.82976 (10)	0.7821 (3)	0.0455 (6)	
N3	0.0669 (3)	0.52930 (11)	0.6995 (3)	0.0597 (7)	
O1	0.7257 (3)	0.71598 (9)	0.7424 (3)	0.0689 (7)	
O2	0.3395 (3)	0.85231 (9)	0.7914 (3)	0.0739 (7)	

### Atomic displacement parameters (Å^2^)

**Table:**

	*U*^11^	*U*^22^	*U*^33^	*U*^12^	*U*^13^	*U*^23^
C1	0.0425 (16)	0.0374 (17)	0.0416 (17)	0.0011 (12)	0.0136 (14)	0.0010 (13)
C2	0.0350 (15)	0.0354 (16)	0.0390 (16)	0.0010 (12)	0.0101 (13)	0.0015 (12)
C4	0.0427 (16)	0.0451 (18)	0.0463 (18)	−0.0018 (13)	0.0209 (14)	−0.0052 (13)
C5	0.0364 (15)	0.0377 (17)	0.0398 (17)	0.0032 (12)	0.0139 (13)	−0.0007 (12)
C6	0.0516 (18)	0.0376 (17)	0.0558 (19)	−0.0034 (14)	0.0236 (16)	−0.0099 (14)
C7	0.0369 (15)	0.0352 (16)	0.0352 (16)	−0.0006 (11)	0.0089 (13)	0.0004 (12)
C8	0.0514 (18)	0.0369 (16)	0.0439 (18)	−0.0014 (14)	0.0162 (15)	−0.0075 (13)
C9	0.0455 (17)	0.0437 (18)	0.063 (2)	−0.0035 (13)	0.0289 (16)	−0.0064 (14)
C10	0.0409 (16)	0.0403 (16)	0.0478 (18)	−0.0064 (12)	0.0180 (14)	−0.0031 (13)
C11	0.0434 (16)	0.0317 (15)	0.0478 (18)	−0.0064 (12)	0.0141 (14)	−0.0004 (13)
C12	0.076 (2)	0.049 (2)	0.096 (3)	−0.0207 (18)	0.044 (2)	−0.0010 (19)
C13	0.0499 (18)	0.063 (2)	0.063 (2)	−0.0012 (15)	0.0082 (17)	0.0014 (17)
C14	0.086 (3)	0.065 (2)	0.068 (2)	−0.0102 (18)	0.049 (2)	−0.0028 (17)
C15	0.117 (3)	0.056 (2)	0.119 (3)	−0.034 (2)	0.090 (3)	−0.033 (2)
C16	0.059 (2)	0.067 (2)	0.089 (3)	−0.0061 (17)	0.021 (2)	−0.036 (2)
C18	0.068 (2)	0.067 (2)	0.081 (3)	0.0073 (18)	0.025 (2)	0.0286 (19)
C19	0.065 (2)	0.0379 (19)	0.106 (3)	−0.0094 (16)	0.036 (2)	−0.0121 (18)
C20	0.138 (4)	0.079 (3)	0.147 (4)	−0.049 (3)	0.110 (4)	−0.036 (3)
N1	0.0433 (14)	0.0372 (14)	0.0532 (16)	−0.0005 (11)	0.0194 (12)	−0.0037 (11)
N2	0.0397 (14)	0.0350 (14)	0.0647 (17)	0.0016 (11)	0.0212 (12)	0.0003 (11)
N3	0.0569 (17)	0.0402 (16)	0.090 (2)	−0.0082 (12)	0.0358 (15)	−0.0159 (13)
O1	0.0569 (14)	0.0418 (13)	0.121 (2)	0.0004 (10)	0.0471 (14)	−0.0134 (12)
O2	0.0542 (14)	0.0416 (13)	0.139 (2)	0.0080 (11)	0.0504 (14)	0.0038 (12)

### Geometric parameters (Å, °)

**Table:**

C1—N3	1.331 (3)	C12—C19	1.343 (4)
C1—C15	1.365 (4)	C12—C20	1.356 (5)
C1—C7	1.486 (4)	C12—H12	0.9300
C2—C6	1.385 (3)	C13—H13A	0.9600
C2—C4	1.394 (4)	C13—H13B	0.9600
C2—C5	1.463 (4)	C13—H13C	0.9600
C4—C8	1.373 (4)	C14—H14A	0.9600
C4—H4	0.9300	C14—H14B	0.9600
C5—N1	1.334 (3)	C14—H14C	0.9600
C5—N2	1.343 (3)	C15—C20	1.387 (5)
C6—C9	1.374 (4)	C15—H15	0.9300
C6—H6	0.9300	C16—H16A	0.9600
C7—C8	1.388 (4)	C16—H16B	0.9600
C7—C9	1.389 (4)	C16—H16C	0.9600
C8—H8	0.9300	C18—H18A	0.9600
C9—H9	0.9300	C18—H18B	0.9600
C10—N1	1.501 (3)	C18—H18C	0.9600
C10—C13	1.517 (4)	C19—N3	1.331 (4)
C10—C14	1.520 (4)	C19—H19	0.9300
C10—C11	1.552 (4)	C20—H20	0.9300
C11—N2	1.497 (3)	N1—O1	1.284 (3)
C11—C16	1.512 (4)	N2—O2	1.274 (3)
C11—C18	1.525 (4)		
			
N3—C1—C15	120.7 (3)	C10—C13—H13B	109.5
N3—C1—C7	116.5 (2)	H13A—C13—H13B	109.5
C15—C1—C7	122.6 (3)	C10—C13—H13C	109.5
C6—C2—C4	118.1 (2)	H13A—C13—H13C	109.5
C6—C2—C5	120.7 (2)	H13B—C13—H13C	109.5
C4—C2—C5	121.2 (2)	C10—C14—H14A	109.5
C8—C4—C2	120.8 (3)	C10—C14—H14B	109.5
C8—C4—H4	119.6	H14A—C14—H14B	109.5
C2—C4—H4	119.6	C10—C14—H14C	109.5
N1—C5—N2	108.7 (2)	H14A—C14—H14C	109.5
N1—C5—C2	126.0 (2)	H14B—C14—H14C	109.5
N2—C5—C2	125.3 (2)	C1—C15—C20	120.1 (3)
C9—C6—C2	120.8 (3)	C1—C15—H15	120.0
C9—C6—H6	119.6	C20—C15—H15	120.0
C2—C6—H6	119.6	C11—C16—H16A	109.5
C8—C7—C9	117.5 (2)	C11—C16—H16B	109.5
C8—C7—C1	120.9 (2)	H16A—C16—H16B	109.5
C9—C7—C1	121.6 (2)	C11—C16—H16C	109.5
C4—C8—C7	121.4 (2)	H16A—C16—H16C	109.5
C4—C8—H8	119.3	H16B—C16—H16C	109.5
C7—C8—H8	119.3	C11—C18—H18A	109.5
C6—C9—C7	121.5 (3)	C11—C18—H18B	109.5
C6—C9—H9	119.3	H18A—C18—H18B	109.5
C7—C9—H9	119.3	C11—C18—H18C	109.5
N1—C10—C13	106.1 (2)	H18A—C18—H18C	109.5
N1—C10—C14	108.6 (2)	H18B—C18—H18C	109.5
C13—C10—C14	109.6 (2)	N3—C19—C12	125.1 (3)
N1—C10—C11	101.6 (2)	N3—C19—H19	117.5
C13—C10—C11	114.5 (2)	C12—C19—H19	117.5
C14—C10—C11	115.6 (2)	C12—C20—C15	118.7 (3)
N2—C11—C16	108.4 (2)	C12—C20—H20	120.7
N2—C11—C18	106.6 (2)	C15—C20—H20	120.7
C16—C11—C18	109.5 (3)	O1—N1—C5	126.4 (2)
N2—C11—C10	101.5 (2)	O1—N1—C10	120.0 (2)
C16—C11—C10	115.7 (2)	C5—N1—C10	113.2 (2)
C18—C11—C10	114.3 (2)	O2—N2—C5	126.2 (2)
C19—C12—C20	117.6 (3)	O2—N2—C11	120.3 (2)
C19—C12—H12	121.2	C5—N2—C11	113.4 (2)
C20—C12—H12	121.2	C19—N3—C1	117.7 (3)
C10—C13—H13A	109.5		
			
C6—C2—C4—C8	1.1 (4)	C7—C1—C15—C20	178.2 (4)
C5—C2—C4—C8	−180.0 (2)	C20—C12—C19—N3	4.3 (6)
C6—C2—C5—N1	152.3 (3)	C19—C12—C20—C15	−3.0 (7)
C4—C2—C5—N1	−26.6 (4)	C1—C15—C20—C12	−0.7 (7)
C6—C2—C5—N2	−26.5 (4)	N2—C5—N1—O1	179.1 (2)
C4—C2—C5—N2	154.6 (3)	C2—C5—N1—O1	0.1 (4)
C4—C2—C6—C9	−0.1 (4)	N2—C5—N1—C10	6.8 (3)
C5—C2—C6—C9	−179.1 (3)	C2—C5—N1—C10	−172.3 (2)
N3—C1—C7—C8	−0.4 (4)	C13—C10—N1—O1	−65.1 (3)
C15—C1—C7—C8	−175.3 (3)	C14—C10—N1—O1	52.6 (3)
N3—C1—C7—C9	178.3 (3)	C11—C10—N1—O1	175.0 (2)
C15—C1—C7—C9	3.4 (4)	C13—C10—N1—C5	107.8 (3)
C2—C4—C8—C7	−1.0 (4)	C14—C10—N1—C5	−134.5 (3)
C9—C7—C8—C4	−0.2 (4)	C11—C10—N1—C5	−12.1 (3)
C1—C7—C8—C4	178.5 (3)	N1—C5—N2—O2	177.4 (3)
C2—C6—C9—C7	−1.0 (4)	C2—C5—N2—O2	−3.6 (4)
C8—C7—C9—C6	1.1 (4)	N1—C5—N2—C11	2.2 (3)
C1—C7—C9—C6	−177.5 (3)	C2—C5—N2—C11	−178.8 (2)
N1—C10—C11—N2	11.7 (2)	C16—C11—N2—O2	52.8 (3)
C13—C10—C11—N2	−102.1 (2)	C18—C11—N2—O2	−65.0 (3)
C14—C10—C11—N2	129.1 (2)	C10—C11—N2—O2	175.1 (2)
N1—C10—C11—C16	128.8 (3)	C16—C11—N2—C5	−131.6 (3)
C13—C10—C11—C16	15.0 (4)	C18—C11—N2—C5	110.5 (3)
C14—C10—C11—C16	−113.7 (3)	C10—C11—N2—C5	−9.4 (3)
N1—C10—C11—C18	−102.6 (3)	C12—C19—N3—C1	−1.6 (5)
C13—C10—C11—C18	143.6 (3)	C15—C1—N3—C19	−2.5 (5)
C14—C10—C11—C18	14.8 (3)	C7—C1—N3—C19	−177.5 (3)
N3—C1—C15—C20	3.5 (6)		

### Hydrogen-bond geometry (Å, °)

**Table:**

*D*—H···*A*	*D*—H	H···*A*	*D*···*A*	*D*—H···*A*
C12—H12···O2^i^	0.93	2.43	3.322 (4)	161

Symmetry codes: (i) −*x*, *y*−1/2, −*z*+3/2.
